# Boron in wound healing: a comprehensive investigation of its diverse mechanisms

**DOI:** 10.3389/fbioe.2024.1475584

**Published:** 2024-10-30

**Authors:** Nasrin Sedighi-Pirsaraei, Amirhossein Tamimi, Faraz Sadeghi Khamaneh, Sana Dadras-Jeddi, Naz Javaheri

**Affiliations:** School of Medicine, Guilan University of Medical Sciences, Rasht, Iran

**Keywords:** wound, chronic wound, wound healing, boron, borate-, boric acid (BA)

## Abstract

Chronic wounds present a significant clinical challenge due to their prolonged healing time and susceptibility to infection. Boron, a trace element with diverse biological functions, has emerged as a promising therapeutic agent in wound healing. This review article comprehensively investigates the mechanisms underlying the beneficial effects of boron compounds in wound healing. Boron exerts its healing properties through multiple pathways, including anti-inflammatory, antimicrobial, antioxidant, and pro-proliferative effects. Inflammation is a crucial component of the wound-healing process, and boron has been shown to modulate inflammatory responses by inhibiting pro-inflammatory cytokines and promoting the resolution of inflammation. Furthermore, boron exhibits antimicrobial activity against a wide range of pathogens commonly associated with chronic wounds, thereby reducing the risk of infection and promoting wound closure. The antioxidant properties of boron help protect cells from oxidative stress, a common feature of chronic wounds that can impair healing. Additionally, boron stimulates cell proliferation and migration, as well as essential tissue regeneration and wound closure processes. Overall, this review highlights the potential of boron as a novel therapeutic approach for treating chronic wounds, offering insights into its diverse mechanisms of action and clinical implications.

## Highlights

• Boron compounds exhibit diverse therapeutic actions in wound healing, including antimicrobial effects, inflammation modulation, oxidative stress reduction, angiogenesis induction, and anti-fibrotic properties.
• Boron’s anti-inflammatory properties play a crucial role in modulating the inflammatory response, promoting the resolution of inflammation, and enhancing the overall healing process.
• Boron’s antimicrobial activity against a wide range of pathogens commonly found in chronic wounds helps reduce the risk of infection and supports wound closure.
• Boron’s antioxidant properties protect cells from oxidative stress, a critical factor in impaired wound healing, promoting tissue regeneration.
• Boron’s ability to stimulate cell proliferation and migration underscores its potential as a novel therapeutic approach for accelerating wound closure and improving clinical outcomes in chronic wound management.
• Boron promotes fibroblast proliferation, migration, and extracellular matrix turnover, leading to faster wound closure with less scar formation.
• Boron has been shown to increase the expression of proteins involved in wound contraction and matrix remodeling, such as collagen, alpha-smooth muscle actin, and transforming growth factor-beta1.

## 1 Introduction

Wounds with aberrations in the healing process are considered chronic wounds (Frykberg and Banks, 2015). Various factors are effectual in wound healing, including local factors, such as oxygenation and infections, and systemic factors, such as age, sex, diseases and drugs (Eming et al., 2007). Diabetic foot ulcers, venous leg ulcers, and pressure ulcers are some examples of the enormous chronic wounds field. Chronic wounds are an ongoing challenge to healthcare systems worldwide (Frykberg and Banks, 2015). For instance, foot ulcers are considerable complications involving one-seventh of diabetics, which have a high recurrence rate and can ultimately lead to amputation. Management of diabetic foot ulcers and amputation cause heavy financial burdens (D-FOOT, 2024). Although various compounds have improved wound healing and reduced scarring, this issue still necessitates more investigations.

Boron is a metalloid and essential trace mineral found in nuts, vegetables, dried fruits, and red wine (Das et al., 2013; Scorei, 2011). It has various industrial, agricultural, nuclear, cosmetic, and medicinal applications (Konca and Korkmaz, 2020; Litovitz et al., 1988; Corradi et al., 2010). Boron plays a vital role in bone formation and maintenance, immune system regulation, antioxidant enzyme secretion, brain function, and cancer prevention and treatment (Das et al., 2013; Scorei, 2011; Konca and Korkmaz, 2020; Pizzorno, 2015; Abdelnour et al., 2018; Jackson et al., 2020; Spears and Armstrong, 2007). It also has beneficial effects in the prevention of epidural fibrosis and the alleviation of side effects of conventional chemotherapies, as well as a significant antimicrobial activity (Das et al., 2013; Pizzorno, 2015; Hazman et al., 2018; Bozkurt et al., 2019; Nzietchueng et al., 2002). Animals treated with boron showed favorable changes in bone density, wound healing, embryonic development, and liver metabolism (Abdelnour et al., 2018). Lack of sufficient boron increases the risk of immunodeficiency, osteoporosis, cancers, arthritis, amnesia, cartilage diseases, hormonal imbalance, and low libido (Scorei, 2011; Abdelnour et al., 2018). On the other hand, excessive amounts of boron can result in cell injury (Abdelnour et al., 2018).

Boron has various medical applications due to its beneficial properties. Boric acid is used to treat yeast vaginitis, and boron compounds are present in multiple medical products (Litovitz et al., 1988; Nzietchueng et al., 2002; De Seta et al., 2009). Boron compounds are used in bacterial antibiotics and targeted therapies for light chain deposition disease and malignancies (Das et al., 2013; Scorei, 2011; Jackson et al., 2020). Boron derivatives are being studied for their potential use in dermatologic disorders and as anti-inflammatory agents for osteoporosis and osteoarthritis (Jackson et al., 2020; Tsubata, 2020). They have also shown promise in the management of dysmenorrhea (Nikkhah and Dolatian, 2020).

As mentioned above, chronic wound remains a challenging issue involving a large population worldwide. Numerous preclinical and several clinical studies have proved the beneficial effects of boron compounds in improving the wound healing process. Moreover, boronic ester bonds with their dynamic properties can add self-healing and environment-responsive features to wound healing formulations. In this study, we concentrated on the mechanisms by which boron influences the wound healing process. Boron-containing compounds used in wound healing include boron-doped ceramics, hydrogels, infused dressings, emulsions, modified silicates, bioactive glasses, boron nanotubes and nanofibers, polysaccharides, and biocomposites. Since there is a wealth of literature in this area, our research does not encompass a clinical or preclinical evaluation of the various boron compounds utilized in wound healing, including their efficacy and chemical properties. We recommend that these aspects be addressed in a separate review article.

## 2 Antimicrobial effects

Infection is one of the major causes of non-healing wounds. The culturable bacteria are more common in chronic wounds than healing wounds. Infection can increase inflammation and fibrosis in the wound and consequently result in a chronic wound (Lv et al., 2022; Scales and Huffnagle, 2013). Bacterial colonization interferes with the healing process of both acute and chronic wounds via breaking epithelium integrity (Scales and Huffnagle, 2013) as well as impairing the function of local cells, including keratinocytes and fibroblasts. In addition, the microbiome affects immune response and the secretion of inflammatory cytokines, such as tumor necrosis factor α (TNF-α), interleukin 6 (IL-6), and interleukin 1beta (IL-1β).

The antimicrobial effects of boron may also contribute to reducing fibrosis in wounds. The microbiome colonized in the wound can influence the function of M2 macrophages residing in the wound site. These macrophages express toll-like receptors (TLRs), which when activated by bacterial pathogen-associated molecular patterns (PAMPs), prompt T cells to release cytokines and chemokines that induce a fibrotic response. Additionally, myofibroblasts, similar to leukocytes, express a range of TLRs, specifically TLRs 2–7. When these TLRs are stimulated, these cells produce chemokines and cytokines, such as CXCL8/IL-8, which attract neutrophils and promote angiogenesis. Additionally, stimulation of TLR9 by bacterial CpG can activate fibrotic pathways. Besides, Inflammatory primary fibroblasts release significant amounts of other chemokines, including CCL5, CCL8, and CXCL6. The increased activation of host cells in a wound, influenced by the microbiota, can negatively impact wound regeneration. Hence, while preventing wound infection, antimicrobial agents may also theoretically benefit wound healing by reducing inflammation at the wound site (Pang et al., 2019; Su et al., 2018). However, it has been shown that the microbiota present in wounds may also provide positive effects on the healing process (Scales and Huffnagle, 2013; Xu and Hsia, 2018). Therefore, the effect of wound microbiota and anti-microbial agents remains controversial in wound healing.

Furthermore, due to the overuse of antibiotics, microbial resistance is a rising issue in global public health (Lv et al., 2022; Scales and Huffnagle, 2013). These agents can't sterilize the wound, and previous studies showed that antibiotic therapy has no positive effect on the healing process of chronic wounds (Scales and Huffnagle, 2013). In this regard, finding new agents and therapeutic manners could prevent microbial colonization and minimize the development of antimicrobial resistance (Lv et al., 2022). The antimicrobial activity is one of the most known properties of boron. Boron has comparable potency with traditional antimicrobial drugs (Iyigundogdu and Saribas, 2022; Fink and Uchman, 2021; Khonina et al., 2020; Kıvanç et al., 2018). No microbial resistance has been reported against boron because the enzymatic system of living organisms is not able to metabolize boron components (Fink and Uchman, 2021). Besides, boron serves as a safe agent regarding cell viability, and it can be used in various forms, such as cream and hydrogel (Kıvanç et al., 2018; Das et al., 2022; Celebi et al., 2024; Temel et al., 2022; Williams, 2020).

### 2.1 Different antimicrobial effects of boron compounds

Previous research indicated that boron components can play an antimicrobial role against bacteria (Celebi et al., 2024; Xue et al., 2013; Wang Y. et al., 2022; Kilic et al., 2020), fungi (Du et al., 2023; Baker et al., 2006; Elewski et al., 2015), and viruses (Windsor et al., 2018; Song et al., 2021). Demirci et al. (2016) showed that both sodium pentaborate pentahydrate (NaB) and boric acid (BA) play antimicrobial roles against bacteria, yeast, and fungi. They found that NaB has a remarkable effect on *staphylococcus aureus* (*S. aureus*), *klebsiella pneumoniae*, *candida albicans,* and *aspergillus niger* infections. Boron’s antibacterial activity is attributed to its reaction with water, which increases free radicals, including hydroxyl radicals, leading to bacterial cell membrane damage (Ghelich et al., 2022; Li et al., 2021; Zhao et al., 2023). On the other hand, a study indicated that hexagonal boron nitride (hBN) does not exhibit antimicrobial properties against *candida albicans* as a yeast, *P. aeruginosa* as a gram-negative bacteria, and *staphylococcus epidermidis* as a gram-positive bacteria in both agar and liquid media (Sen et al., 2019).

Hydrogels are widely used for treating wounds. Recently, Boron has been a part of the composition of these products. A boron-based probe driven theranostic hydrogel was developed for visual monitoring and matching chronic wounds’ healing (Zhang et al., 2023). Zhang et al. (2023) expressed that a boron-based (BT) probe, made of borax (B) and tannic acid (TA), serves as a cross-linker for constructing the *guar gum/polyvinyl alcohol/BT* (GPBT) hydrogels that can disrupt bacterial membrane. Furthermore, *bioactive silicon–boron-containing glycerohydrogel* has been shown to have bactericidal activity against *Streptococcus pyogenes (S. рyogenes)*, *methicillin-resistance S. аureus* (MRSA), *P. aeruginosa* and *escherichia coli* (*E. coli*) as well as fungistatic activity against *Trichophyton rubrum* (*Tr. rubrum*) and *Tr. violaceum* (Chupakhin et al., 2017). Table 1 presents studies evaluating the antimicrobial properties of boron compounds against common wound pathogens. Only studies that clearly identified the microorganisms tested are included.

**TABLE 1:** Antimicrobial effects of boron-containing compounds against microorganisms commonly associated with wound infections.

No	Compound	Author	Fungi	Bacteria	Mechanism of action	Method
1	sodium pentaborate pentahydrate (NaB)	Demirci et al. (2016)	*Candida albicans, Aspergillus niger*	*S. aureus* and *Klebsiella pneumoniae*	NR	*In vitro*
2	boric acid (BA)	Sayin et al. (2016)	NR	*S. aureus* and *Vibrio anguillarum*	bacteriostatic and bactericide	*In vitro*
		Celebi et al. (2024)	NR	*P. aeruginosa, Klebsiella pneumoniae, Enterococcus faecalis, S. aureus, MRSA, E. coli, MRCNS, Enterobacter aerogenes, and Acinetobacter baumannii*	Inhibition of biofilm formation	*In vitro*
3	disodium octaborate tetrahydrate (DOT)	Sayin et al. (2016)	NR	*S. aureus* and *Vibrio anguillarum*	bacteriostatic and bactericide	*In vitro*
4	SiO_2_-Na_2_O-CaO-B_2_O_3_-P_2_O_5_	Datta et al. (2018)	NR	*E. coli*	NR	*In vitro*
5	SiO_2_-CaO-B_2_O_3_-ZnO (SCBZ)	Mehrabi and Mesgar (2022)	NR	*S. aureus and E. coli*	NR	*In vitro*
6	boron-complexed polyglycerol–chitosan dendrimers ((PGLD-Ch)B)	de Queiroz et al. (2006)	NR	*S. aureus* and *P. aeruginosa*	interaction of cationic (PGLD-Ch)B with the electronegative surface of bacterial cells, resulting in cell membrane disruption	*In vitro*
7	bioactive silicon–boron-containing glycerohydrogel	Chupakhin et al. (2017)	*Trichophyton rubrum (Tr. rubrum)* and *Trichophyton violaceum (Tr. Violaceum)*	*S. рyogenes,* MRSA*, P. aeruginosa* and *E. coli*	NR	*In vitro*
8	Alpha lipoic acid (ALA) conjugated hBN nanoparticles (NPs)	Türkez et al. (2022)	NR	*S. aureus* and *E. coli*	NR	*In vitro*
9	polyvinylpyrrolidone (PVP) nanofibers in combination with boron	Ghelich et al. (2022)	NR	*S. aureus*	Rupture of cell membrane because of boron’s reaction with water that results in increasing free radicals, including OH	*In vitro*
10	polyvinylpyrrolidone (PVP) nanofibers in combination with boron and hafnium (Hf)	Ghelich et al. (2022)	NR	*S. aureus* and *E. coli*	Cell membrane damage because of boron reaction with water that results in increasing free radicals, including OH	*In vitro*
11	boron-dipyrromethene (BODIPY)	Li et al. (2021)	NR	*S. aureus* and *E. coli*	Cell membrane damage and inhibiting bacterial doubling by producing ROS and NO accompanied by PDT	*In vitro* and *in vivo*
12	Positively charged boron-dipyrromethene (LIBDP)	Shi et al. (2023)	NR	*S. aureus*	Bacteriostatic through cell membrane damage and inhibition of biofilm formation	*In vitro* and *in vivo*
13	porphyrin and BODIPY-based covalent organic framework (COF) called Tph-BDP-COF	Zhao et al. (2023)	NR	*E. coli*	Damage to cell membrane and intracellular proteins via ROS production and photothermal conversion capability	*In vitro*
14	*Boron nanosheet (B NS)-coated quaternized chitosan (QCS) and NO donor N,N′-di-sec-butyl-N,N′-dinitroso-1,4-phenylenediamine (BNN6) (B-QCS–BNN6)*	Lv et al. (2022)	NR	*MRSA, S. aureus* and *E. coli*	Cell membrane damage because of photothermal conversion and NO release	*In vitro* and *in vivo*
15	Nano-MgB2	Meng et al. (2022)	NR	*P. aeruginosa*, *S. aureus*	Cell membrane damage and inhibition of bacteria-induced inflammation via trapping peptidoglycans and lipopolysaccharides accompanied by the production of metal cations and an alkaline microenvironment and changing the expression of genes related to protein export, aminoacyl-tRNA biosynthesis, and ribosomes	*In vitro* and *in vivo*
16	poly (lactic acid) (PLA) based nanofibrous PLA-OMMT/ZB composites	Dilmani et al. (2023)	NR	*S. aureus* and *E. coli*	Bactericide: Quaternary ammonium salts like TMOD can modify the permeability of cell membranes Bacteriostatic: due to the presence of zinc particles in fibrous structures	*In vitro*
17	poly (lactic acid) (PLA) based nanofibrous PLA-OMMT/PBA composites	Dilmani et al. (2023)	NR	*S. aureus* and *E. coli*	Bactericide: Quaternary ammonium salts like TMOD can modify the permeability of cell membranes	*In vitro*
18	poly (lactic acid) (PLA) based nanofibrous PLA-OMMT/BN composites	Dilmani et al. (2023)	NR	*S. aureus*	Bactericide: Quaternary ammonium salts like TMOD can modify the permeability of cell membranes	*In vitro*
19	boron doped graphdiyne nanosheet (B-GDY)	Bi et al. (2022)	NR	*S. aureus* and *E. coli*	provoking peroxidase enzyme, producing ROS, and rupturing cell membrane	*In vitro* and *in vivo*
20	the *guar gum/polyvinyl alcohol/boron based-2* (GPBT) hydrogel	Zhang et al. (2023)	NR	*MRSA, S. aureus* and *E. coli*	bacteriostatic effect by the damage caused by the introduction and release of tanic acid on the bacterial membrane	*In vitro*
21	*chitosan (CS) membrane loaded with copper boron–imidazolate framework* (Cu–BIF)	Wang et al. (2022b)	NR	*S. aureus* and *E. coli*	Cell membrane damage	*In vitro*
22	calcium metaborate (CMB)	Celebi et al. (2024)	NR	NR	Inhibition of biofilm formation	
23	sodium metaborate tetrahydrate (SMTB)	Celebi et al. (2024)	NR	*P. aeruginosa*, *Klebsiella pneumoniae*, *Enterococcus faecalis*, *S. aureus,* MRSA, *E. coli*, MRCNS, *S. hemolyticus*, *Enterobacter aerogenes*, and *Acinetobacter baumannii*	Inhibition of biofilm formation	*In vitro*
24	zinc borate (ZB)	Celebi et al. (2024)	NR	*NR*	Inhibition of biofilm formation	*In vitro*
25	sodium tetrafluor borate (STFB)	Celebi et al. (2024)	NR	*P. aeruginosa*, *Klebsiella pneumoniae*, *Enterococcus faecalis*, *S. aureus,* MRSA, *E. coli*, MRCNS, *S. hemolyticus*, *Enterobacter aerogenes*, and *Acinetobacter baumannii*	Inhibition of biofilm formation	*In vitro*
26	sodium tetraborate (STB)	Celebi et al. (2024)	NR	*P. aeruginosa*, *Klebsiella pneumoniae*, *Enterococcus faecalis*, *S. aureus,* MRSA, *E. coli*, MRCNS, *S. hemolyticus*, *Enterobacter aerogenes*, and *Acinetobacter baumannii*	Inhibition of biofilm formation	*In vitro*
27	potassium tetrafluoroborate (PTFB)	Celebi et al. (2024)	NR	*NR*	Inhibition of biofilm formation	*In vitro*
28	ammonium pentaborate tetrahydrate (APTB)	Celebi et al. (2024)	NR	*NR*	Inhibition of biofilm formation	*In vitro*
29	sodium perborate monohydrate (SPM)	Celebi et al. (2024)	NR	*Klebsiella pneumoniae*, *Enterococcus faecalis*, *S. aureus,* MRSA, *E. coli*, MRCNS, *S. hemolyticus*, *Enterobacter aerogenes*, and *Acinetobacter baumannii*	Inhibition of biofilm formation	*In vitro*
30	Borax (STBD)	Celebi et al. (2024)	NR	*P. aeruginosa*, *Klebsiella pneumoniae*, *Enterococcus faecalis*, *S. aureus,* MRSA, *E. coli*, MRCNS, *S. hemolyticus*, *Enterobacter aerogenes*, and *Acinetobacter baumannii*	Inhibition of biofilm formation	*In vitro*
31	ammonium tetrafluorine borate (ATFB)	Celebi et al. (2024)	NR	*P. aeruginosa*, *Enterococcus faecalis,* MRSA, *E. coli*, *S. hemolyticus*, *Enterobacter aerogenes*, and *Acinetobacter baumannii*	Inhibition of biofilm formation	*In vitro*
32	Potassium metaborate	Celebi et al. (2024)	NR	*P. aeruginosa*, *Klebsiella pneumoniae*, *Enterococcus faecalis*, *S. aureus,* MRSA, *E. coli*, MRCNS, *S. hemolyticus*, *Enterobacter aerogenes*, and *Acinetobacter baumannii*	Inhibition of biofilm formation	*In vitro*

NR, Not Reported; S. рyogenes, Streptococcus pyogenes; S. hemolyticus, Staphylococcus hemolyticus; MRSA, Methicillin-Resistance S. Aureus; P. aeruginosa, Pseudomonas aeruginosa; E. coli, Escherichia coli; S. aureus, Streptococcus aureus; MRCNS, methicillin-resistant coagulase-negative Staphylococcus; OH, Hydroxyl radicals; NO, nitric oxide; ROS, reactive oxygen species; PDT, photodynamic therapy; PTT, photothermal therapy; TMOD, trimethyl octadecyl ammonium bromide.

### 2.2 Antibiofilm activity

Biofilm formation is suggested to participate in drug resistance, which is a growing issue in wounds (Pang et al., 2019). *Pseudomonas aeruginosa* (*p. aeruginosa*) clusters gather together and release a polymeric matrix that combines with the extracellular matrix (ECM) and necrotic tissue to create a biofilm (Pang et al., 2019). These biofilms promote quorum sensing (QS), a process that regulates the expression of virulence genes in various microorganisms. The QS system triggers the bacteria to produce virulence factors once their population reaches a certain threshold, eliminating polymorphonuclear leukocytes (PMNs). Boron compounds have shown promise in preventing biofilm formation (Sayin et al., 2016).

Celebi et al. (2024) recently studied the antimicrobial effects of different boron compounds against biofilm-forming bacteria obtained from blood cultures. They examined the minimal inhibition concentration (MIC) values of calcium metaborate (CMB), sodium metaborate tetrahydrate (SMTB), zinc borate (ZB), sodium tetrafluor borate (STFB), sodium tetraborate (STB), potassium tetrafluoroborate (PTFB), ammonium pentaborate tetrahydrate (APTB), sodium perborate monohydrate (SPM), Borax (STBD), Potassium metaborate and ammonium tetrafluorine borate (ATFB). The inhibitory effect of boron compounds against different biofilm-forming pathogens is shown in Table 1. All the boron derivatives exhibited an inhibitory effect against biofilm formation after 4 h in the infected fibroblast cells, but bacterial growth increased rapidly after 18 h. Bacterial resistance, the disappearance of substance activity, or toxicity of these compounds to fibroblasts after 4 h are suggested as the probable mechanisms for the rebound bacterial growth.

### 2.3 Synergic antimicrobial effects of boron with other compounds

Several studies have demonstrated that boron, in combination with other components, can benefit wound healing through its considerable antimicrobial function. Sayin et al. (2016) reported that BA and disodium octaborate tetrahydrate (DOT) exhibited bactericidal and bacteriostatic effects against different bacteria, along with inhibiting biofilm inhibition. They showed bacteriostatic activity against *S. aureus,* and *P. aeuroginosa* was among the most sensitive strains to boron compounds (Sayin et al., 2016). In addition, Boron oxide (B_2_O_3_) and its combination with compounds such as SiO_2_-Na_2_O-CaO-B_2_O_3_-P_2_O_5_ and SiO_2_-CaO-B_2_O_3_-ZnO (SCBZ) can restrict the activity of *S. аureus* and *E. coli* (Datta et al., 2018; Mehrabi and Mesgar, 2022). Moreover, *boron-complexed polyglycerol–chitosan dendrimers* ((PGLD-Ch)B) can inactive *S. аureus* and *P. aeruginosa* by inhibiting their proliferation. They proposed two main mechanisms for the antibacterial properties of (PGLD-Ch)B. The first involved the transport of boron from the compound. The second mechanism was due to the cationic nature of (PGLD-Ch)B, which facilitates its interaction with the electronegative surface of bacterial cells, potentially disrupting the outer membrane barrier of Gram-negative bacteria (de Queiroz et al., 2006). In addition, Wang and Zhenga showed that a *chitosan (CS) membrane loaded with copper boron–imidazolate framework* (Cu–BIF) could significantly improve wound healing in bacterial-infected wounds (Wang G. et al., 2022).

### 2.4 Antimicrobial effects of boron as a photosensitizer

Recently, phototherapy in the form of photothermal therapy (PTT) and photodynamic therapy (PDT) has frequently been utilized for bacterial eradication via photosensitizers (PSs) that can produce reactive oxygen species (ROS). Several studies have used various forms of boron-dipyrromethene (BODIPY) in phototherapy. Shi et al. (2023) indicated that BNP1-BNP3 improves wound healing and exerts a bacteriostatic activity against *staphylococcus aureus* by BODIPY self-assembly. Its bacteriostatic activity has been suggested to be caused by destroying the cell membrane and inhibiting biofilm formation. BNP2 also inhibits the excessive inflammation in infected wounds. Additionally, Li et al. (2021) reported that BODIPY containing a guanidine group LIBDP has antibacterial activity and annihilates pre-formed biofilm via destroying bacterial membrane and inhibiting bacterial doubling by producing ROS and nitric oxide (NO). Moreover, Zhao et al. (2023) demonstrated that porphyrin and BODIPY-based covalent organic framework (COF) called Tph-BDP-COF has a substantially more potent antibacterial activity in comparison with porphyrin-based COF-366 through its superior ROS production and photothermal conversion capability.

### 2.5 Antimicrobial effects of nano forms of boron

Boron has also been used in nano forms as an antimicrobial agent in phototherapy. *Boron nanosheet (B NS)-coated quaternized chitosan (QCS) and NO donor N,N′-di-sec-butyl-N,N′-dinitroso-1,4-phenylenediamine (BNN6) (B-QCS–BNN6)* are used in PTT and can also elevate NO release after laser stimulation. The combination of PTT and NO release can inactivate more than 99.9% gram-negative and gram-positive bacteria during 5 min and promote the healing process in wounds infected by MRSA (Lv et al., 2022).

Moreover, literature has shown that boron nanoparticles (NPs) are effective in wound healing through their antimicrobial activity. Metal boride nanoparticles (MB NPs) such as Nano-MgB_2_ can enhance bacterial death without promoting bacteria-induced inflammation. It is hydrolyzed to create boron dihydroxy groups for trapping peptidoglycans and lipopolysaccharides. The function of boron dihydroxy groups is accompanied by the production of metal cations and an alkaline microenvironment. All the mentioned mechanisms damage the bacterial membrane. The antimicrobial effect of Nano-MgB_2_ in *P. aeruginosa* was comparable with gentamicin, amikacin, ciprofloxacin, levofloxacin, and better than imipenem, meropenem, ceftazidime, cefepime, ampicillin-sulbactam, aztreonam. In Nano-MgB_2_-treated P*. aeruginosa,* genes related to the ribosome, aminoacyl-tRNA biosynthesis, and Protein export pathways were upregulated (Meng et al., 2022). Regarding *S. aureus*, the antibacterial activity of the mentioned NPs was superior to that of cephalexin but not as effective as erythromycin and mupirocin. In another study, Türkez et al. (2022) evaluated the antimicrobial activity of alpha lipoic acid (ALA) conjugated hBN and boron carbide (B_4_C) nanoparticles (NPs). The results demonstrated that ALA-conjugated hBN NPs improve wound healing and act as antimicrobial agents against *S. aureus* and *E. coli* with a higher potential than ALA-B_4_C. However, hBN, ALA-B_4_C, and ALA compounds all offered favorable regenerative and antimicrobial effects.

In previous literature, boron has also been used as a part of nanofibers to heal wounds. Boron-containing nanofibers can suppress external inflammation secondary to microbial infections (Sen et al., 2019). Dilmani et al. (2023) used boron nitride (BN), zinc borate (ZB), phenylboronic acid (PBA), and Organo-modified Montmorillonite (OMMT) to produce poly (lactic acid) (PLA) based nanofibrous PLA-OMMT/B composites. They reported that the mixture of ZB and PBA showed significant bacteriostatic and bactericidal activity against gram-negative and gram-positive bacteria. The mixture exerted its effects through the alteration of the permeability of the cell membrane and the presence of zinc in its fibrous structure. In addition, Ghelich et al. (2022) showed that polyvinylpyrrolidone (PVP) nanofibers in combination with boron or/and hafnium (Hf) exhibit antibacterial effects against *S. aureus* and *E. coli*. Their suggested mechanism for these effects was cell membrane rupture. The membrane damage results from boron’s reaction with water that increases free radicals, including hydroxyl radicals (OH), which can result in phospholipid peroxidation. Meanwhile, the electron deficiency of B^3+^ leads to an increase in OH− and surface Lewis acid, which ultimately results in cell membrane rupture by capturing electron-hole pairs and converting them into free (·OH) groups.

Nanozymes having enzyme-like activity can serve as potent bactericidal agents. Boron has an important role in the proper functioning of these agents. In a study, Bi et al. (2022) demonstrated that boron-doped graphdiyne nanosheet (B-GDY), provoking peroxidase (POD) enzyme, can produce ROS and therefore destroy gram-positive and gram-negative bacteria and enhance wound healing.

## 3 Inflammation modulation

Wound healing is a complex process involving a series of events from initial injury to scar tissue formation, influenced by various internal and external factors (Gethin, 2012). Hemostasis and inflammation are the primary steps in wound healing (Guo and DiPietro, 2010). The body’s immediate response to injury is acute inflammation, a crucial phase that triggers the release of growth factors and signaling molecules to guide the repair process. This begins when platelets encounter exposed collagen, forming a blood clot. Along with neutrophils, these platelets release factors such as platelet-derived growth factor (PDGF) and transforming growth factor-beta (TGP-b) that amplify the clotting process and attract other cells involved in inflammation. As platelets aggregate, clotting factors are released, forming a fibrin clot that acts as a temporary scaffold at the injury site. The initial inflammatory response aims to clear damaged tissue and debris, typically lasting about 2 days. Although inflammation is necessary for wound healing by removing microorganisms, prolonged inflammation leads to healing impairment and can result in the development of chronic wounds (Demirci et al., 2016; Guo and DiPietro, 2010). In chronic wounds, this phase can become prolonged, leading to excessive breakdown of the extracellular matrix, hindering proper healing (Gethin, 2012; Eming et al., 2007; Diegelmann and Evans, 2004; Szpaderska et al., 2003). Recent studies indicated that boron affects inflammation by changing the expression and translation of genes and proteins involved in the inflammation process. Herein, we summarized the reported mechanisms underlying the effects of boron derivatives administration.

### 3.1 Inflammatory cell infiltration

Inflammatory cells such as mast cells, eosinophils, neutrophils, macrophages, and lymphocytes play a crucial role in regulating the inflammation process (Wang Z. et al., 2022; Nagata et al., 2020). Neutrophils are among the first to arrive at an injury site, peaking in numbers within 24 h. They engulf and destroy foreign material, bacteria, and damaged cells and matrix components. They also produce human neutrophil elastase (HNE), an enzyme that breaks down fibronectin, contributing to the problems of chronic wounds (Gethin, 2012). Damaging of mast cells result in releasing granules containing histamine and other chemicals, causing blood vessels to become more permeable, allowing immune cells to reach the injury site (Gethin, 2012). Macrophages, derived from monocytes, become active in wound healing after 48 h. They clean up debris and bacteria, release growth factors to attract fibroblasts and smooth muscle cells, and regulate the breakdown of damaged tissue. Their presence signals the end of the inflammatory phase and the beginning of the proliferative phase (Gethin, 2012; Schultz et al., 2005).

Konca and Korkmaz (2020) showed that oral and topical boron consumption can promote inflammatory cell formation in rats. In contrast, an *in vivo* study showed that nano-MgB_2_ can reduce neutrophil and macrophage emigration induced by dead bacteria (Meng et al., 2022). In addition, Büyük et al. (2021) compared inflammatory cell infiltration between fito and boron groups in full-thickness wound models of rats. The results illustrated that inflammatory cell infiltration was lower in the boron group.

### 3.2 Protease inhibition

Proteases, particularly matrix metalloproteinase (MMPs) and serine proteases, are essential for normal wound healing. However, if their elevated levels persist, it can lead to the formation of chronic wounds. They cause excessive breakdown of the extracellular matrix. This disrupts cell migration, weakens the tissue, and contributes to chronic wounds. Therefore, proteases and their inhibitors play an essential role in maintaining a balance between breaking down and building up the ECM, which is crucial for the effective and coordinated healing of skin wounds (McCarty and Percival, 2013). Boron may ameliorate excessive inflammation by inhibiting proteases (Smoum et al., 2012). Boron derivatives modulate the turnover of extracellular matrix components by promoting their release from cells, reducing their production, and enhancing the activity of intra- and extracellular proteases. Specifically, alkenyl phosphonoboronates have shown moderate inhibitory effects on MMP-2 (Smoum et al., 2012).

MMP inhibitors are crucial in regulating protease activity, but their deficiency can lead to uncontrolled tissue breakdown. Chronic wounds often exhibit lower MMP levels compared to acute wounds, and specific MMPs contribute to skin damage in certain conditions like venous ulcers. Pergament et al. (2002) developed phosphonoboronates that showed moderate inhibitory effects on MMP-2. They suggested zinc chelation and transition state analog mechanisms as possible mechanisms for this inhibitory effect. On the other hand, boron can increase the intracellular levels of MMP-2 and 9 in keratinocytes *in vitro*, which is essential for wound healing (Chebassier et al., 2004a). Taken together, these findings reveal that boron’s influence on MMPs’ activity is complex and needs further clarification.

Activated leukocytes release serine proteases, a sub-group of proteolytic enzymes, including elastase, chymase, and cathepsin G that play pivotal roles in the inflammatory response (Hunt and Idso, 1999; Fritz et al., 1986; Niehaus et al., 1990). BA and substituted boric acid compounds have been shown to inhibit serine protease activity by binding to the active site of the enzyme, thus inhibiting excessive inflammation (Abdelnour et al., 2018; Durick et al., 2005). In a study, Nzietchueng and colleagues indicated that BA does not change trypsin-like and collagenase activity but can inhibit elastase *in vitro* investigations (Nzietchueng et al., 2002). However, in an *in vivo* examination of fibroblasts, they found that BA enhanced cathepsin D, collagenase, and trypsin-like activity, while boron reduced alkaline phosphatase activity in human serum (Nzietchueng et al., 2002). In addition, Benderdour et al. (1998a) demonstrated that protease activity increases following BA administration in cartilage and culture medium. Overall, these results indicate that the impacts of boron on proteolytic enzymes are different based on the type of enzyme. It can enhance the activity of some enzymes, while it can inhibit or have no effect on the level of others.

### 3.3 Soluble protein mediators

Three main types of soluble proteins, growth factors, cytokines, and chemokines, play crucial roles in wound healing. Cytokines, produced by various cells, including inflammatory cells, keratinocytes, and fibroblasts, can promote or suppress inflammation. In addition, they regulate the activity of non-inflammatory cells, such as the production of MMPs by fibroblasts, which are essential for tissue remodeling (Gethin, 2012). Boron compounds have shown regulatory effects on these proteins.

Durick et al. (2005) evaluated boron’s impact on the NF-κB (nuclear factor kappa light chain enhancer of activated B cells) pathway-regulated proteins, including IL-1β, TNF-α, macrophage inflammatory protein-1 alpha (MIP-1a) and inducible nitric oxide synthase (iNOS). They compared the expression of the mentioned proteins between two groups: LPS-activated murine macrophages in the absence of boron and LPS-stimulated murine macrophages grown in 10 or 100-mmol supplemental boron. The results showed that the level of the mentioned factors decreased in the boron-containing group. In contrast, the superoxide dismutase (SOD) transcription rate, which is not under the control of the NF-κB pathway, had no change (Durick et al., 2005). Hence, boron has the potential to inhibit the inflammation process by acting at a site prior to the activation of cytokine genes in the pathway regulated by NF-kB.

BA elevates TNF-α and heat-shock proteins 70 that are related to wound healing. TNF-α is one of the cytokines regulated by the transforming growth factor beta (TGF-β) signaling pathway. It is necessary for inflammatory response, fibroblast growth induction, macrophage infiltration, and angiogenesis. Moreover, the secretion of other wound healing-related cytokines increases following the fibroblast growth stimulation (Benderdour et al., 1998a; Benderdour et al., 1997; Doğan et al., 2014; Spears and Armstrong, 2007). NaB is another boron component that, in combination with pluronic F68 and F127, could elevate the level of TNF-α (Doğan et al., 2014). On the other hand, Büyük et al. (2021) also indicated that fibroblast proliferation and TNF-α expression were lower following treatment with boron. Moreover, Benderdour and colleagues revealed that low-dose boron derivatives, including acyclic-amine carboxy boranes, boronated pyrimidines, and purine and tripeptide-boranes, could have anti-inflammatory impacts in rodents. They found that these compounds ameliorate pain by suppressing lysosomal hydrolytic enzyme and reducing TNF-α secretion (Benderdour et al., 1998a; Hall et al., 1996; Hall et al., 1995). Therefore, the effects of boron compounds on the expression and secretion of TNF-α remain controversial and need further clarification.

In another investigation, Celebi et al. (2022) examined BA and potassium metaborate’s impacts on the interleukin change in a fibroblast wound model in cultural media. They evaluated interleukin levels in the *MRSA*-infected group compared to the control group. The results indicated that the level of inflammatory cytokines, including IL-1β and IL-110, which increased in the *MRSA* group, was reduced with BA and potassium metaborate administration. The expression of IL-10 as an anti-inflammatory cytokine was approximately equivalent in the *MRSA* group to the control group after BA treatment. Also, potassium metaborate can enhance IL-10 levels nearly 1-fold compared with the control group (Celebi et al., 2022). In addition, López-Cabrera studied the effects of boron-containing compounds on diabetic rats (López-Cabrera et al., 2018). The experimental diabetes model was induced by combining a diet high in fat with multiple low doses of streptozotocin. The result indicated that IL-6 level, as an inflammatory biomarker, was lower in boric acid, cyclohexylboronic acid, and normal chow-diet groups than in the high-fat diet and streptozotocin group. However, the groups had no significant difference in C-reactive protein (CRP) levels (López-Cabrera et al., 2018).

Dead tissue in a wound provides a breeding ground for bacteria, increasing the workload for immune cells and delaying healing. While controlled breakdown of the extracellular matrix is crucial for wound repair, excessive breakdown caused by bacteria can lead to chronic inflammation and hinder the progression to the next healing phase (Gethin, 2012). Boron has also been used as a nano-drug to regulate inflammation caused by bacteria. To illustrate, Meng et al. (2022) demonstrated that nano-MgB_2_ not only has no toxic effects on keratinocytes and immunocytes but can also reduce the expression of proinflammatory proteins. In this investigation, macrophages were stimulated by dead bacteria or dead bacteria-related endotoxin (LPS). These agents can increase the level of inflammatory molecules, including TNF-α, interleukin-6 (IL-6) and IL-1β (Spears and Armstrong, 2007). LPS can induce inflammatory factors through toll-like receptor 4 activation and regulation of the mitogen-activated protein kinase (MAPK) signaling pathway (Frazier et al., 2012; Wang et al., 2017). However, nano-MgB_2_ administration significantly diminished their expression in media in the study of Meng et al. (2022). The mentioned study expressed that nano-MgB2 considerably decreases p38, Erk, and JNK, which are the results of MAPK pathway phosphorylation. Besides, *in vivo* examination indicated that nano-MgB_2_ could hinder LPS-induced inflammation. In fact, nano-MgB_2_ inhibits the lipogenesis and hematoxylin-positive cells, which are increased in the dermis during inflammation, presenting with redness and swelling. TNF-α, IL-6, and monocyte chemoattractant protein-1 (MCP-1) were also inhibited by nano-MgB_2_ (Meng et al., 2022).

### 3.4 Nitric oxide reduction

Nitric oxide (NO) is an essential part of the inflammatory process. NO, produced by various cells involved in wound healing, plays a multifaceted role in the repair process. Its production, regulated by nitric oxide synthases (NOSs), is influenced by inflammatory signals. NO’s actions contribute to inflammation, blood vessel formation, and cell growth, all of which are tightly controlled by specific cytokine networks (Schwentker et al., 2002). NO exerts its effects through various mechanisms. Some involve its chemical reaction with oxygen to generate reactive species, while others relate to its interaction with iron-containing enzymes like guanyl-cyclase (Witte and Barbul, 2002). Demirci et al. (2016) investigated boron derivatives’ anti-inflammatory effects on mouse macrophages. The results demonstrated that a high dose (200 µg) of BA and NaB can significantly reduce the NO level. Additionally, the study displayed that even low doses of boron can inhibit the expression of two major genes related to inflammation mediators, cyclooxygenase-2 (COX-2) and iNOS (Demirci et al., 2016). Suppression of iNOS hinders collagen production, reduces the strength of incision wounds, and impairs healing in wound models (Witte and Barbul, 2002). Accordingly, although boron may benefit wound healing by ameliorating inflammation, it may also impair healing through several mechanisms. A deeper understanding of these interactions is needed to determine whether the benefits of boron’s anti-inflammatory effects outweigh its potential detrimental impacts.

In addition, Scorei and Rotaru mentioned that calcium fructoborate plays an anti-inflammatory role in culture media by suppressing superoxide anions, IL-6, IL-1β, and NO secretion and increasing TNF-α expression (Scorei and Rotaru, 2011). Moreover, TNF-α can induce the production of anti-inflammatory cytokines and certain acute-phase proteins, which are able to scavenge oxygen radicals and inhibit serine protease. This ultimately leads to wound healing facilitation (Spears and Armstrong, 2007; Scorei and Rotaru, 2011). Boron can also suppress inflammatory response through lowering oxidative stress, which has been discussed in the next part of the article with details.

## 4 Oxidative stress reduction

Free radicals, produced as a byproduct of cellular processes, are harmful to cells and can damage important molecules. While they contribute to inflammation by attracting immune cells, they can also cause damage to DNA, lipids, and other cell components (Gethin, 2012). Reactive oxygen species (ROS) are necessary to protect the wound from external injuries, such as bacteria, and serve as secondary messengers in some cellular pathways, such as BCR signaling, MAPK/ERK, PTK/PTP, and PI3K-AKT-mTOR pathway (Tsubata, 2020; Checa and Aran, 2020). However, an inordinate amount of them restricts wound healing due to reducing anti-oxidant components (Guo and DiPietro, 2010; Dunnill et al., 2017; Cano Sanchez et al., 2018). To illustrate, the diabetic wound healing restriction is partly related to the extreme oxidizing environment that is contributed to hyperglycemia and tissue hypoxia (Dworzański et al., 2020; Deng et al., 2021).

### 4.1 ROS reduction

Anti-oxidant agents play a crucial modulatory role in wound healing by removing ROS. Some of these agents include thioredoxin (Trx), superoxide dismutase (SOD), glutathione (GSH)‐related glutathione s‐transferases (GSTs), glutathione peroxidases (GPx), catalase (CAT), UDP‐glucuronosyl transferases (UGTs), epoxide hydrolase, NADP(H) quinone oxidoreductase (NQO1), heme oxygenase‐1 (HO‐1) and glutamylcysteine synthetase (Dunnill et al., 2017).

Several studies showed that boron can reduce ROS and improve the function of anti-oxidants, while no significant change was observed in culture media with some boron compounds, including hBN, hBN-ALA, B_4_C, B_4_C-ALA (Türkez et al., 2022). Geyikoglu et al. (2019) reported that BA alone or in combination with propolis (prop) works as an anti-oxidant agent in ovarian ischemia-reperfusion (I/R) injury by reducing the level of myeloperoxidase (MPO), IL-6 and malondialdehyde (MDA) and the expression of 8-hydroxylo-2′-deoxyguanosin (8-OHdG), TNF-α and caspase-3. Another investigation on the antioxidant properties of BA showed that boron protects renal tissue from I/R injury via increasing SOD, CAT, and GSH and decreasing MDA and total oxidant status (TOS) (Kar et al., 2020). Boron also preserves brain, liver, and gastric tissue from ethanol-induced oxidative stress (İlhan et al., 2023). Boron supports gastric tissue by alleviating ROS, MDA, IL-6, TNF-α, and JAK2/STAT3 action, as well as improving AMPK activity (Gündoğdu et al., 2023). In addition, Calcium fructoborate, a boron derivative, has been shown to play an anti-oxidant role by scavenging superoxide ions (Scorei and Rotaru, 2011). Moreover, Tepedelen et al. (2016) investigated the protective role of BA in epithelial cells. They expressed that BA can decline DNA damage caused by irinotecan (CPT-11), doxorubicin (Doxo), etoposide (ETP), and H2O2 and facilitate wound healing.

Glutathione (GSH), a vital antioxidant, is found in high concentrations in living organisms, protecting cells from oxidative damage. However, GSH can hinder the antibacterial activity of many nanozymes that produce ROS (Wang et al., 2020; Wu et al., 2019). Therefore, the ability to function effectively in a GSH-rich environment is crucial for antibacterial nanozymes. A Boron-doped graphdiyne nanosheet (B-GDY) demonstrated exceptional capability in this regard (Bi et al., 2022). While control groups failed to consume GSH, B-GDY not only withstood high concentrations of GSH but also actively depleted it within 1 hour. This unique ability highlights B-GDY’s capacity to overcome the inhibitory effects of GSH and maintain its antibacterial activity (Bi et al., 2022).

Moreover, Nicotinamide adenine dinucleotide phosphate (NADPH) is another vital molecule involved in a wide range of cellular processes, including antioxidant systems and electron transport. Its concentration significantly impacts cellular metabolism. A study showed that B-GDY can efficiently oxidize NADPH. This highlights the potential of B-GDY to influence cellular metabolism by directly affecting NADPH levels (Bi et al., 2022).

NAD+, NADP−, and FAD-requiring oxidoreductase enzymes are the regulators of wound healing. For instance, xanthine oxidoreductase (XOR), an enzyme known for its involvement in the purine metabolic pathway, is a potential generator of ROS in the wound environment. It catalyzes the conversion of hypoxanthine to xanthine and then xanthine to uric acid using NAD + or O2 (Fernandez et al., 2018). An *in vitro* study indicated that BA and substituted boric acid compounds, suppressing these enzymes, can act as prominent regulatory factors in inflammation (Durick et al., 2005). However, regarding the anti-oxidant impact of boron on wound healing, Tarhan and colleagues indicated that the polydopamine coating of hBN (hBN@pdopa) does not remarkably alter ROS levels in human umbilical vein endothelial cells (HUVECs), while high doses of the silver nanoparticles (AgNPs) decorated on hBN@pdopa subsides ROS levels (Tarhan et al., 2021). Therefore, the anti-oxidant properties of boron remain controversial and may depend on its combination with other elements.

### 4.2 Lowering DNA oxidation

Regarding the protecting effects of BA against oxidative stress, Celebi et al. (2022) showed that 8-OHdG reduces following BA and potassium metaborate administration in *MRSA*-infected fibroblast culture. Considering that 8-OHdG is one of the principal DNA oxidation products, using boron can have a remarkable effect in reducing oxidative stress (Celebi et al., 2022). Moreover, Coban et al. (2015) demonstrated that administration of boron can decline the oxidative activity of malathion in a dose-dependent manner in mice via elevating the level of 8-OHdG and MDA.

### 4.3 Inhibiting lipid peroxidation

Konca and Korkmaz evaluated the effects of different types of boron administration, including oral (OB), local (LB), and the combination of oral and local (OLB) on full-thickness skin lesions (Konca and Korkmaz, 2020). They observed that MDA levels, as an important biomarker of oxidative stress, were considerably reduced in OB and OLB groups in comparison with the control group. Lower MDA levels illustrated the suppression of lipid peroxidation and elimination of free oxygen radicals. In diabetic patients, hyperglycemia elevates lipid peroxidation products and free irons in plasma, resulting in impaired angiogenesis and wound healing. Imperfect angiogenesis is due to the inhibition of vascular endothelial growth factor (VEGF), expression (Feng et al., 2022; Altavilla et al., 2001). Therefore, boron may improve wound healing by hindering lipid peroxidation and increasing the level of VEGF. Furthermore, they also reported that the GSH level was increased in OB and OLB groups, which can clear away free oxygen radicals from the tissues. SOD was elevated in LB and OLB groups, while it was lowered in the control group. Fujiwara and colleague (Fujiwara et al., 2016) showed that reduction of SOD level as an anti-oxidant agent is associated with impaired wound healing in aged mice. Hence, boron may improve the wound-healing process through SOD augmentation. CAT rose in OLB, LB, and OB groups, unlike the control one (Konca and Korkmaz, 2020). This enzyme is a peroxisomal enzyme that can reduce H_2_O_2_ concentration by converting it to water and molecular oxygen (Abdel-Mageed et al., 2012).

## 5 Angiogenesis induction

Blood vessels supply oxygen and substantial nutrients, and the formation of new blood vessels is a pivotal part of wound healing along with the migration of keratinocytes and fibroblasts (Tonnesen et al., 2000; Schugart et al., 2008). Accordingly, impaired neovascularization is a common defect in the acute and chronic wounds (Demirci et al., 2016; King et al., 2014). Therefore, finding an efficient treatment and preventing it from getting worse will help to heal the wounds.

### 5.1 Angiogenic cytokine secretion

Angiogenesis and fibroblast function depend on the presence of specific growth factors and macrophage-provided cytokines. Several studies have shown the remarkable effects of boron on angiogenesis through increasing cytokine release and endothelial cell proliferation (Dousset et al., 2002; Ahtzaz et al., 2020; Kapukaya and Ciloglu, 2020; Oğur et al., 2021; Hu et al., 2018; Mehrabi et al., 2022). It has been shown that boron can boost the secretion of granulocyte-macrophage colony-stimulating factor (GM-CSF) and Granulocyte colony-stimulating factor (G-CSF). Previous studies have reported that these cytokines are the probable pro-angiogenetic factors that function via JAK2 phosphorylation and STAT-3 activation. In addition, angiogenesis is induced by VEGF and basic fibroblast growth factor (bFGF) (Aliper et al., 2014; Zhao et al., 2011). Zhao and colleagues showed that suppressing JAK2 and inhibiting STAT3 activation decreases the expression of VEGF and bFGF (Zhao et al., 2011). Considering all the above, the effect of boron on the JAK2/STAT3 signaling pathway during the wound healing process needs further clarification with more studies.

It has been reported that BA and NaB induce the secretion of G-CSF, GM-CSF, fibroblast growth factor-7 (FGF-7), and TGF-B1 (Demirci et al., 2016). Bruno and colleagues studied the combination of GM-CSF and G-CSF administration in acute myocardial infarction (AMI) patients. Their results indicated that endothelial progenitor cells (CD14^+^/KDR^+^) increased in peripheral blood sample of the patients following the treatment with GM-CSF and G-CSF in comparison with patients who had not received the mentioned intervention (Bruno et al., 2006). Therefore, it can be suggested that boron may have the potential to enhance angiogenesis through the elevation of GM-CSF and G-CSF levels. In addition, Demirci et al. (2016) found that NaB can elevate TGF-B3 level (Benderdour et al., 1998b). TGF-B1 and TGF-B3 improve neovascularization (Pakyari et al., 2013).

Investigations have suggested that boron can change the expression of particular genes, followed by altered RNAs and protein synthesis. It induces VEGF secretion followed by angiogenesis (Benderdour et al., 1998b). Dzondo-Gadet et al. (2002) showed that BA considerably accelerates the synthesis of RNAs, including RNAs associated with angiogenesis-related proteins, such as VEGF and TGF-B. However, they have not detected any change in the level of RNAs related to TGF-1 and TNF-α (Dzondo-Gadet et al., 2002). A study showed that HUVECs in a boron-rich culture media produce higher levels of bFGF and IL-6, which act as pro-angiogenetic cytokines (Haro Durand et al., 2014). Boron concentration in their study was higher than normal plasma levels but lower than the toxic dose.

### 5.2 Eph/ephrin pathway

A transmembrane protein called Eph receptor, binding to ephrin ligands, can be detected in various organs. These proteins form a subfamily of receptor tyrosine kinases. They play a crucial role in the regulation of arteriovenous endothelial cell formation, stem cell differentiation, migration of intestinal epithelial cells, immune system response, bone remodeling, and angiogenesis (Büyük et al., 2021; Kuijper et al., 2007; Zhang and Hughes, 2006). In this regard, Büyük et al. (2021) investigated Eph/ephrin pathway activity after boron, fito, and pluronic gel administration to diabetic wounds and compared it with a control group in rats. The results showed that ephrinA1, ephrinB1, and ephrinB2 expression and EphB4 immunoreactivity were higher in the boron group compared to fito, pluronic, and control groups. Therefore, the development of endothelial cell formation and angiogenesis can be considered following the ability of boron to increase the expression of the mentioned components and, subsequently, Eph–ephrin signaling activation.

### 5.3 Anti-angiogenic effects

Demirci et al. (2016) showed an anti-angiogenic role of boron derivatives. They found that high doses of BA and NaB can limit the growth of endothelial cells, restricting the quantity and extent of developing vessels in a dose-dependent manner (Demirci et al., 2016). Sen et al. (2019) reported that the neovascularization process can be influenced by environmental agents, including extra-cellular matrix, cultural media, and their interaction with endothelial cells. They observed that endothelium growth progressed in hBN-containing media, while there was no significant growth in BA-containing media (Sen et al., 2019). Hence, studies have shown conflicting results about the effect of boron components on angiogenesis, which requires further investigation.

## 6 Antifibrotic effects

Adult mammals respond to injury by prioritizing rapid tissue closure. This involves accelerated fibroblast growth and excessive production of extracellular matrix, which leads to scar formation and functional impairment of the repaired tissue. While many promising growth factors and other agents have been identified to promote tissue regeneration, their clinical application is hampered by several challenges, including difficulties in maintaining their bioactivity within injured tissues due to poor retention, limited tissue penetration, and the instability of protein therapeutics in the presence of proteases (Järvinen and Ruoslahti, 2010). In addition to the several important benefits of boron compounds in different stages of wound healing, they have also shown promising anti-fibrotic effects that can be favorable for certain clinical circumstances. Borax, a sodium salt of boron, has been shown to remarkably improve cardiac function and diminish apoptosis and myocardial fibrosis following myocardial infarction in rats (Bouchareb et al., 2020). Moreover, In a randomized animal study, Bozkurt and colleagues revealed that boric acid can significantly lower post-laminectomy epidural fibrosis in rats (Bozkurt et al., 2019). It was also reported that the Cu-BIF/CS membrane not only resulted in a smaller area of unhealed skin but also encouraged the alignment of collagen fibers along the direction of tension. This facilitated wound contraction and promoted proper skin tissue remodeling and maturation (Wang G. et al., 2022), Considering the challenges and serious consequences of epidural fibrosis, this can be a fruitful feature of boron compounds, but it needs further evaluation, especially by clinical studies.

Boron-containing hydrogels have recently been widely used as wound dressings. In this regard, Hua et al. (2023) designed a hydrogel using boron in the form of gelatin methacryloyl phenylboronic acid/cis-diol-crosslinked (GMPD) that can be used following urethral reconstruction for healing without scars. With the severe burden of urethral strictures on patients’ quality of life, this may offer a notable option for several urologic procedures. Boron’s antifibrotic feature may also prevent adhesion bands following abdominal surgeries, which needs further clarification by future studies. In addition, (Zhang et al., 2023) showed that the guar gum/polyvinyl alcohol/borax/tanic acid (GPBT2) hydrogels can monitor wound PH and repair it *in vitro* and *in vivo*. After 14 days of treatment with GPBT2, they conducted a histological analysis of the wound tissue to examine the development of skin appendages, collagen fibrin deposition, and scar tissue. The control and GPB groups showed fewer hair follicles, sebaceous glands, and collagen fibers than the GPBT2 hydrogel group, which displayed a clear presence of hair follicles, sebaceous glands, and well-organized collagen fibers. Furthermore, the scar width in the GPBT2 hydrogel group was significantly narrower compared to both the control and GPB groups, with the GPB group also showing a notable reduction in scar width compared to the control group. In another study, a silicon-boron glycerohydrogel exhibited promising effects in reducing the severity and depth of skin damage (Chupakhin et al., 2017).

## 7 Granulation tissue formation and Re-epithelialization

The formation of a complete epithelial layer signals the successful completion of wound healing. Many boron compounds, such as Cu-BIF/CS membrane (Wang G. et al., 2022), GPBT2 hydrogel (Zhang et al., 2023), bioactive silicon–boron-containing glycerohydrogel (Chupakhin et al., 2017) and NaB-containing hydrogel (Demirci et al., 2016) have accelerated this stage even more than 30%, resulting in a complete and healthy epidermis along with mild fragmentation. The suggested mechanism for GPBT2 hydrogel activity involves reversing the inflammatory microenvironment and enhancing angiogenesis via down-regulating the JAK/STAT pathway. Then, the suppression of STAT1 is associated with the M2 macrophage polarization and, following that, cytokine release, angiogenesis, and improvement of the wound healing (Zhang et al., 2023).

### 7.1 Keratinocyte migration

Epithelialization of the wound is an essential part of wound healing by reconstructing the cutaneous barriers. It involves the migration and proliferation of keratinocytes. Boron compounds have been shown to facilitate epithelialization in various ways (Chebassier et al., 2004b). In a study by Chebassier et al. (2004b) keratinocytes incubated with boron salts for 24 h have shown significantly accelerated wound closure. The effect of boron on wound closure was not related to the proliferation of the keratinocytes, suggesting that boron affects wound healing through the migration of keratinocytes.

### 7.2 Fibroblast proliferation and migration

Fibroblasts are pivotal cells in wound healing, playing a crucial role in producing collagen and other extracellular matrix components, including fibronectin, glycosaminoglycans, proteoglycans, and hyaluronic acid, which are vital for tissue repair and regeneration (Tottoli et al., 2020). These cells are active in different phases of wound healing. By increasing the synthetic activity of fibroblasts, Boron improves extracellular-matrix turnover, which in turn may result in faster and better wound healing with less scar formation. Boron compounds have shown favorable effects on fibroblasts’ proliferation, migration, and activity (Pizzorno, 2015). Gundogdu et al. (2020) have demonstrated that nanoemulsion formulations containing boron offer beneficial impacts on fibroblast proliferation. The same results were reported for the combination gel treatment containing NaB and Pluronics hydrogel (Doğan et al., 2014) and also both BA and NaB-containing hydrogels at intermediate concentrations (up to 200 μg/mL) during the first 24 h of incubation (Demirci et al., 2016). Besides, diabetic rat wound examination showed that NaB-Gel treatment reduced inflammation, increased fibroblast density, better granulation tissue formation, and improved epithelial integrity. While hydrogel treatment also showed improvements, NaB-Gel demonstrated a more significant reduction in inflammation and a greater increase in collagen deposition and fibroblast density (Demirci et al., 2016).

Moreover, Datta et al. (2018) used NIH3T3 mouse embryonic fibroblast cell line to assess the *in vitro* cell proliferation in bulk glass samples over 3, 5, and 7, using base glass (BG0B) as a control. The results indicated that the BG1B sample, containing B2O3 substituting 25% of SiO2, exhibited the highest level of cell proliferation. In contrast, cell proliferation decreased progressively with increasing B2O3 content in BG2B, BG3B, and BG4B glass samples. Notably, their BG4B sample, which only contained B2O3, showed slightly lower cell proliferation than the base glass.

Additionally, another study showed a significantly higher expression of the Akt gene, which is a gene related to cellular proliferation and growth, using boron (Demirci et al., 2015). Besides, multiple studies have reported that using boron compounds results in higher migration of human dermal fibroblasts, which was associated with faster wound closure (Gundogdu et al., 2020; Demirci et al., 2015). In one study, boron-containing hydrogel has been associated with higher migration of fibroblasts (Demirci et al., 2015). It also led to an increased expression of proteins related to the contraction of the wound, including collagen, α-smooth muscle actin, TGF-β1, vimentin, and vascular endothelial growth factor. The increased migration is due to enhanced matrix metalloproteinase expression. It is part of an essential effect of boron in wound healing, increasing extracellular matrix turnover (Demirci et al., 2015).

### 7.3 Extracellular-matrix formation

Studies have also shown that boron promotes proteoglycan, collagen, and protein synthesis (Nzietchueng et al., 2002; Benderdour et al., 1997; Benderdour et al., 1998b). Nzietchueng et al. (2002) found that Boron can increase trypsin-like, collagenase, and cathepsin D activities in fibroblasts *in vivo*, which contradicts the result of *in vitro* studies of the same researchers. They noted a direct inhibitory effect of boron on elastase and alkaline phosphatase activities. They also suggested that since boron increases phosphorylation, parts of its products may be indirect mediators involved in wound healing, especially TNF (Nzietchueng et al., 2002).

**FIGURE 1:**
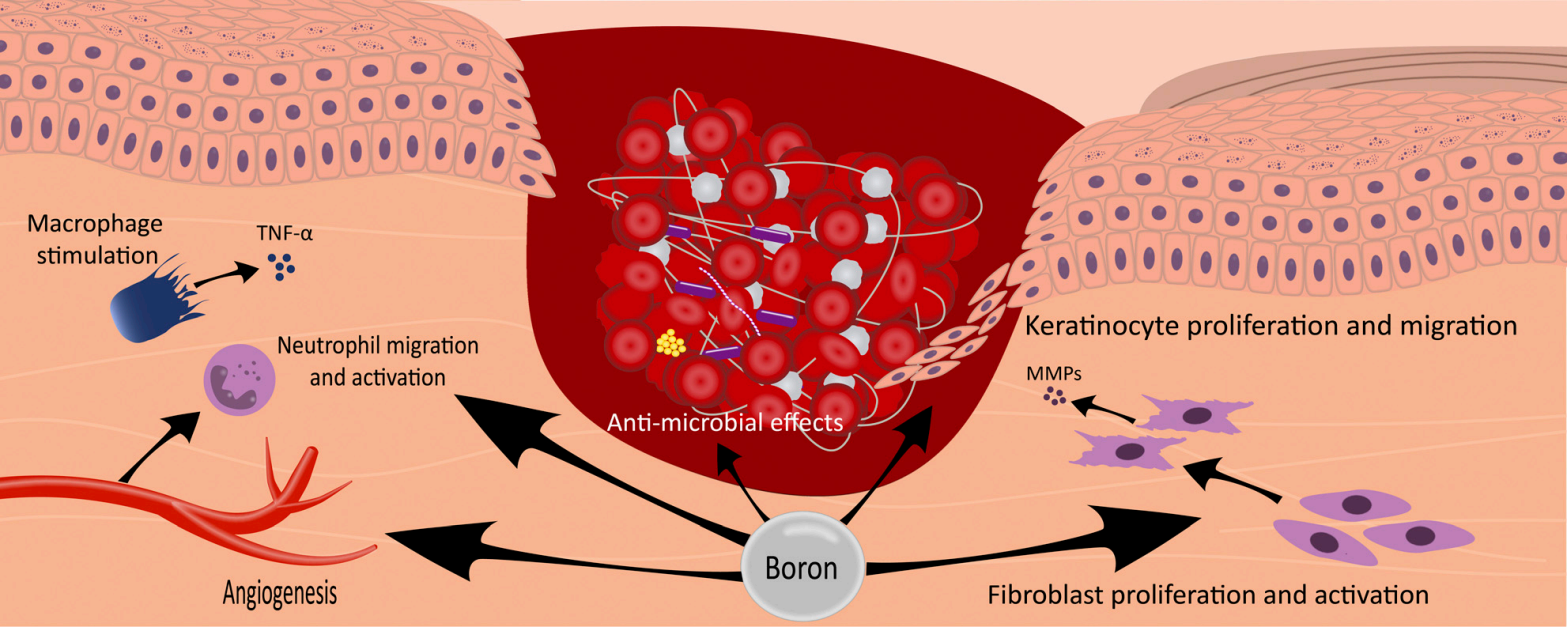
The illustration depicts the multifaceted effects of boron in the wound-healing process. Boron compounds exhibit a range of therapeutic actions, including the promotion of angiogenesis, stimulation of macrophages with subsequent secretion of TNF-alpha, facilitation of neutrophil migration and activation, enhancement of fibroblast proliferation and activation, stimulation of keratinocyte proliferation and migration, and exertion of antimicrobial effects. These diverse mechanisms contribute to boron interventions’ overall accelerated wound healing.

Moreover, in another study, NaB treatment significantly increased the expression of fibronectin and laminin genes in Human fibroblast cells in a dose-dependent manner, while BA had no notable effect. However, both BA and NaB significantly enhanced the expression of these genes in dermal HaCaT and L-929 cells (Demirci et al., 2016). In addition, it has been shown that the combination treatment with a hydrogel containing NaB (boron source) and pluronics (F68 and F127) can significantly promote collagen production in wound tissue (Doğan et al., 2014). Different potential effects of boron on wound healing are presented in Figure 1.

### 7.4 Infected wound closure

The re-epithelization of infected wounds is one of the challenges in the wound healing process. In a study, Meng et al. (2022) mentioned that bacterial infections damage epidermal epithelial cells and elevate lipogenesis and hematoxylin-positive cells in the dermis, suggesting an inflammatory response. Following treatment with Nano-MgB2, the healing of the epidermis was expedited, and there was a marked reduction in both lipogenesis and hematoxylin-positive cells in the dermis, suggesting a decreased inflammation. Additionally, the granulation tissues transformed into scar tissue with a higher presence of fibroblasts, indicating that Nano-MgB2 facilitated wound repair in infected skin.

In another study, Lv et al. (2022) compared re-epithelialization between four groups, including B-QCS, BNN6, B-QCS–BNN6 + NIR, and an untreated group. In the group treated with B-QCS-BNN6 and near-infrared (NIR) light, the reconstructed skin tissue displayed a thicker, well-formed epithelial layer with evenly distributed collagen fibers, indicating proper tissue repair. Furthermore, skin appendages like hair follicles began to migrate towards the wound edges, signifying a more complete and natural healing process. Masson’s trichrome staining highlighted a significant increase in collagen deposition in the B-QCS-BNN6 + NIR group compared to other groups. This further demonstrates that combining B-QCS-BNN6 and NIR light effectively accelerates the re-epithelialization of bacterial-infected wounds, promoting a faster and more complete recovery. Moreover, Wang et al. reported that The Cu–BIF/CS membrane has the potential to enhance wound healing speed and promote skin regeneration by utilizing a combination of chitosan, Cu^2+^, and hydroxyl radicals during the treatment of infected wounds.

Furthermore, Bi et al. (2022) declared that B-GDY significantly accelerated wound healing in a rat model infected with *S. aureus*. Along with its antimicrobial effects, the combination of B-GDY and H_2_O_2_ led to reduced inflammation, faster re-epithelialization, and enhanced collagen formation, resulting in nearly complete wound closure within 9 days.

## 8 Side effects

Before using boron to treat wound infection, it is essential to evaluate its biological toxicity to ensure that it is non-toxic to the tissues. While high doses of systemic boron consumption can be fatal, the exact toxic dose varies depending on the specific boron compound, the cells, and the administration type. Therefore, the safe and effective dose of boron remains a subject of ongoing research (Dilmani et al., 2023).

### 8.1 Topical administration side effects

While our research has not identified any serious adverse effects associated with topical boron administration, a limited number of *in vitro* studies have observed cytotoxic effects from certain boron compounds. The application of Cu-BIF/CS membranes in cell cultures showed a minor reduction in cell viability, attributed to the slow release of copper ions and the inherent biocompatibility of chitosan. Moreover, there was no evidence supporting the injury of the heart, liver, spleen, lung, or kidney following topical boron administration in cutaneous wound models. Therefore, the use of this membrane for the treatment of wound infections is highly promising (Wang G. et al., 2022). Similarly, bioactive silicon–boron-containing glycerohydrogel showed low systemic toxicity as well as when applied topically (Chupakhin et al., 2017). However, the systemic absorption of topical boron needs further clarification.

#### 8.1.1 Toxicity to fibroblast

As mentioned, human dermal fibroblast (HDFa) cells play a prominent role in wound healing. The toxicity of boron and its toxic dose varies depending on the type of boron derivative. Doğan et al. (2014) showed that NaB enhances the viability of HDF cells, particularly at a concentration of 15 μg/mL, especially during the first 3 days of treatment. On the other hand, in another study, higher concentrations of BA and NaB at above 500 μg/mL have shown cytotoxic effects after 72 h in dermal cells, including HDF, HaCaT, L-929, and HUVEC (Demirci et al., 2016).

Bi et al. (2022) investigated the B-GDY’s safety by examining its effects on mouse fibroblast cells and its compatibility with blood. B-GDY demonstrated low toxicity to fibroblast cells, with over 85% viability even at high concentrations. Furthermore, it exhibited excellent blood compatibility, showing minimal hemolysis at concentrations up to 100 μg/mL. Additionally, *in vivo* experiments also did not show any injury to major organs, including the heart, liver, lung, kidney, and spleen. In addition, Türkez et al. (2022) evaluated the effect of hexagonal boron nitride (hBN), boron carbide (B_4_C), hBN-ALA, B4C- alpha lipoic acid (ALA), and ALA in wound healing. They reported that hBN-ALA and B4C-ALA nanoparticles exhibited low levels of cytotoxicity on the HDFa cell line, with no difference compared with controls in concentrations of 50 μg/mL and lower. These concentrations even enhanced cell viability compared to the control group. Notably, when comparing the B4C and B4C-ALA groups to the controls, it was observed that the addition of ALA significantly diminished the cytotoxicity of B4C on the HDF cell line.

Moreover, Dilmani et al. (2023) examined the toxicity of PLA-OMMT/B at a concentration of 0.04 g/mL on fibroblasts in 3 3-day period. The OMMT-containing nanofibers showed moderate cytotoxicity on HDF cells on the second day, likely due to the presence of TMOD. Moreover, the rate of cell growth rose until the third day, suggesting these matrices are suitable for short-term wound dressing applications. The result was similar to another study that used quaternary ammonium salts at concentrations up to 0.5 g/mL over a 3-day period (Sha et al., 2021). Table 2 presents studies evaluating the cytotoxic effects of topical boron compounds.

**TABLE 2:** Review of the reported toxicity of topical boron derivatives in the wound treatment.

	Boron compound	Author	Study type	Cell/animal type	Therapeutic dose	Side effect		Toxicity mechanism
						Local	Systemic	
1	Cu-BIF/CS membranes	Wang et al. (2022b)	*In vitro* and *in vivo*	Mouse	NR	L	No	NR
2	bioactive silicon–boron-containing glycerohydrogel	Chupakhin et al. (2017)	*In vivo*	NR	NR	No	No	NR
3	B-GDY	Bi et al. (2022)	*In vitro* and *in vivo*	Mouse embryonic fibroblast	100 μg/mL	L	No	NR
4	hBN	Türkez et al. (2022)	*In vitro*	Human dermal fibroblast	50 μg/mL	No	NR	NR
5	B_4_C	Türkez et al. (2022)	*In vitro*	Human dermal fibroblast	50 μg/mL	No	NR	NR
6	hBN-ALA	Türkez et al. (2022)	*In vitro*	Human dermal fibroblast	50 μg/mL	No	NR	NR
7	B_4_C-ALA	Türkez et al. (2022)	*In vitro*	Human dermal fibroblast	50 μg/mL	No	NR	NR
8	PLA-OMMT/B composites	Dilmani et al. (2023)	*In vitro*	Human dermal fibroblast	0.04 g/mL	L	NR	Altering the permeability of cellular membranes due to the Presence of TMOD in the OMMT structure
9	NaB	Doğan et al. (2014)	*In vitro* and *in vivo*	Human fibroblast	15 μg/mL	No	No	NR
		Demirci et al. (2016)	*In vitro* and *in vivo*	Human fibroblast, HaCaT, L-929 and HUVEC/Rat	200 μg/mL	No	NR	No
10	BA	Demirci et al. (2016)	*In vitro* and *in vivo*	Human fibroblast, HaCaT, L-929, and HUVEC/Rat	200 μg/mL	No	NR	No
11	Nano-MgB2	Meng et al. (2022)	*In vitro*	keratinocyte or immunocyte	100 μg/mL	No	NR	NR
12	B-QCS–BNN6	Lv et al. (2022)	*In vitro*	MCF-7 and HeLa cell lines	500 μg/mL	L	NR	The combined effects of photothermal and NO therapy
13	GPBT2 hydrogel	Zhang et al. (2023)	*In vitro*	Mouse fibroblast cells	100 mg/mL	L	NR	NR

NR, Not Reported; L, Low toxicity; GPBT, The guar gum/polyvinyl alcohol/boron-based; B-QCS–BNN6, Boron nanosheet (B NS)-coated quaternized chitosan (QCS) and NO donor N,N′-di-sec-butyl-N,N′-dinitroso-1,4-phenylenediamine (BNN6); BA, Boric Acid; NaB, Sodium pentaborate pentahydrate; PLA, Poly (lactic acid); B_4_C-ALA, Alpha lipoic acid (ALA)-boron carbide (B_4_C); hBN-ALA, alpha lipoic acid (ALA) conjugated hexagonal boron nitride (hBN); B_4_C, Boron Carbide; hBN, hexagonal boron nitride; B-GDY, boron doped graphdiyne nanosheet; Cu-BIF/CS, chitosan (CS) membrane loaded with copper boron–imidazolate framework (Cu–BIF); TMOD, trimethyl octadecyl ammonium bromide.

#### 8.1.2 Toxicity to keratinocytes

Keratinocytes, principal cells of the skin, contribute significantly to wound healing, not only as structural cells but also by performing essential immune functions, interaction with fibroblast, and re-epithelialization through their migration, proliferation, and differentiation (Werner et al., 2007; Piipponen et al., 2020; Raja et al., 2007). Given that, Meng et al. (2022) reported that after 24 h of incubation, Nano-MgB2 did not exhibit any toxicity to skin keratinocytes or immunocytes. Additionally, the substances released from Nano-MgB2, including Mg^2+^ and H_3_BO_3_, were non-toxic to keratinocytes.

#### 8.1.3 Toxicity to other cell types

Breast cancer cell line MCF-7 and cervical cancer cell line Hela were used to examine the toxicity of the B-QCS–BNN6 boron derivative. Lv et al. (2022) evaluated the viability of the cells co-cultured with B-QCS–BNN6 in different concentrations. The B-QCS-BNN6 material demonstrated excellent biocompatibility and was well-tolerated by cells, showing over 90% cell viability in both HeLa and MCF-7 cells. This high level of cell viability was maintained even at a concentration of 500 μg/mL after 24 h of incubation. Moreover, B-QCS-BNN6 did not cause any noticeable tissue damage or inflammation, suggesting that it is unlikely to have any significant toxic effects. However, cell viability was decreased when exposed to an 808 nm laser for 5 min. This is attributed to the combined impact of photothermal and NO therapy, both known to have cytotoxic effects. They also evaluated the material’s blood compatibility through hemolysis testing. B-QCS-BNN6 exhibited low hemolysis rates below 5% across different concentrations, indicating excellent blood compatibility. Even after photothermal therapy, the material’s blood compatibility remained favorable. This demonstrates that B-QCS-BNN6 possesses sufficient safety for potential clinical applications.

### 8.2 Systemic administration side effects

Boron is a trace element, usually found at a concentration of 0–0.2 mg/dL in plasma with a half-life of 5–10 h, and 1–2 mg of it is needed in the daily diet (Pizzorno, 2015; Edwall et al., 1979). Symptomatic boron intoxication has been reported to be infrequent and rarely may lead to significant adverse effects and death. Boron can cause a range of symptoms, such as headaches, hypothermia, restlessness, dermatitis, and anorexia, with the severity depending on the dose and individual factors (Dilmani et al., 2023). In a study by Litovitz et al., in 784 cases of acute boron ingestion, 88.3% remained asymptomatic (Litovitz et al., 1988). In other patients, gastrointestinal-related symptoms like vomiting, nausea, and abdominal pain occurred most frequently. Less common symptoms included lethargy, headache, light-headedness, seizure, skin rash, and fever (Litovitz et al., 1988; Corradi et al., 2010). They also reported one death after boron ingestion. Additionally, boric acid intoxication may also lead to hypotension, metabolic acidosis, acute renal failure, and shock (Corradi et al., 2010).

The absorption of Boric acid varies significantly in different parts of the gastrointestinal tract. In mouth mucosa, with or without gingivitis and periodontitis, its absorption is insignificant, but in other parts of the GI tract, it increases up to approximately 100% (Corradi et al., 2010; Edwall et al., 1979). Following boric acid ingestion, its concentration surges in the blood with a peak of 2 h. Therefore, early hemodialysis may be the most effective therapeutic option for boric acid intoxication. However, hemodialysis only in the first 12 h after ingestion could cause a decline in mortality rate. In addition, about 20% of blood-entered boric acid cannot be detoxified by dialysis due to protein bindings. Hence, adding forced diuresis to the treatment, such as furosemide and intravenous fluid administration, can improve the result of therapy (Corradi et al., 2010; Teshima et al., 2001). In a study by Litovitz et al., symptomatic cases of boric acid ingestion underwent hemodialysis. They showed that this therapy can cause a reduction in the boric acid serum half-life and clinical symptoms of the patients. However, still, most cases do not benefit from this aggressive treating (Litovitz et al., 1988).

## 9 Conclusion

In conclusion, Boron compounds have the potential to revolutionize wound care and provide new solutions to the ongoing challenge of chronic wounds worldwide. They can accelerate wound healing at a low cost and with high efficacy, while maintaining acceptable safety profiles. Their antimicrobial and anti-oxidant properties are particularly significant for treating burn and diabetic wounds, and their angiogenic effects are especially beneficial for diabetic patients. Furthermore, these compounds have demonstrated favorable antifibrotic activity, which can be advantageous in post-surgical recovery. In this study, we focused on the mechanisms through which boron impacts the wound healing process. Given the extensive literature on this subject, our research does not include a clinical or preclinical evaluation of the different boron compounds employed in wound healing, nor does it assess their efficacy and chemical properties. We recommend that these essential aspects be explored in a separate review article.
